# Supplementary material for: TAS2R38 taster variants-linked MGAM expression in Alzheimer’s disease: a novel target for precision drug repurposing
Source: Front Aging Neurosci. 2026 Mar 4;18:1768436. doi: 10.3389/fnagi.2026.1768436 (PMC12996105; doi:10.3389/fnagi.2026.1768436)
Supplement: Supplementary file 1 [file Data_Sheet_1.pdf]

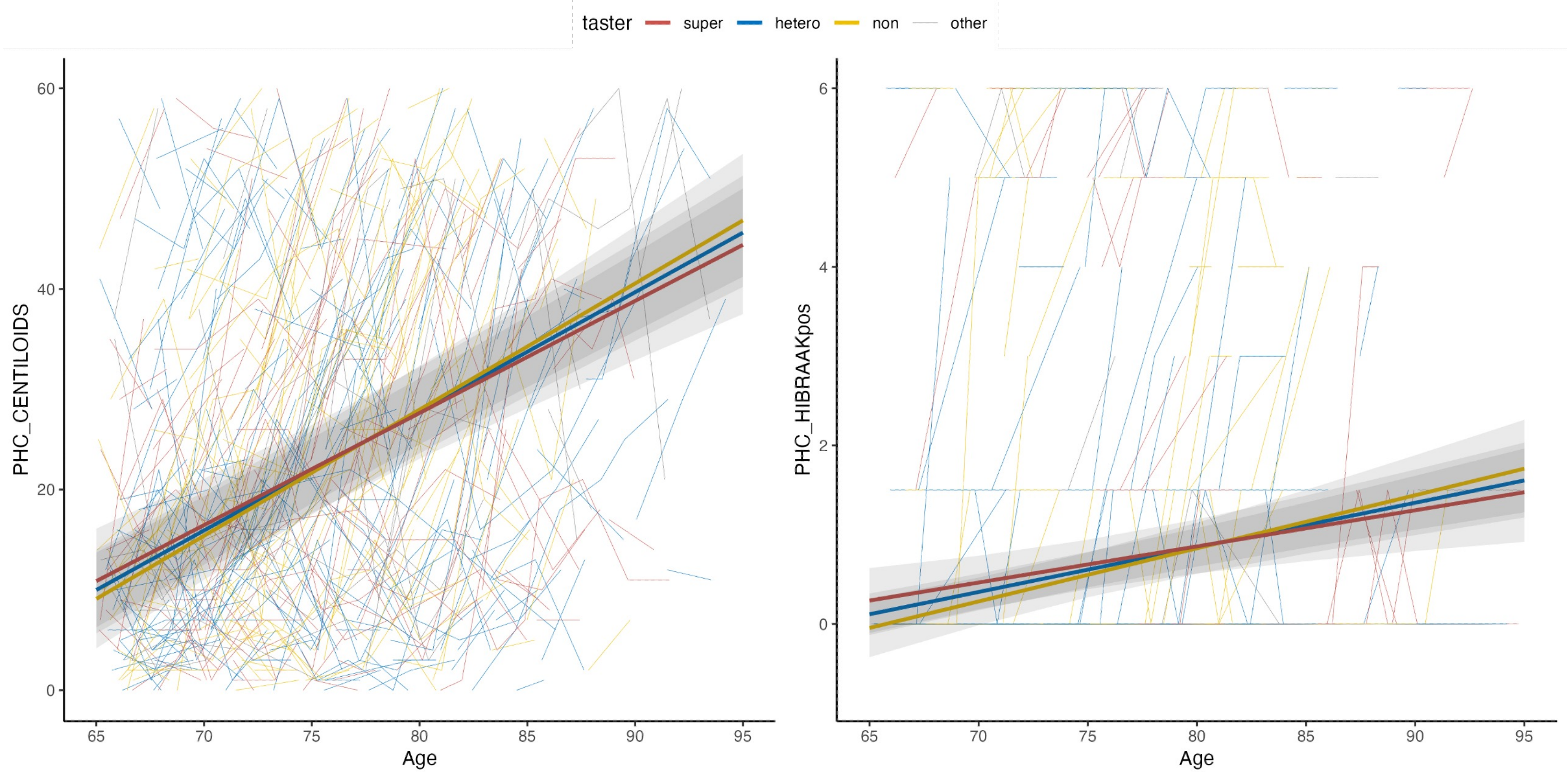

Supplementary Figure 1: Longitudinal changes in PET imaging biomarkers (amyloid and tau), with fitted lines stratified by different taster groups in ADNI. In addition to WGS data, additional genetic data were obtained from ADNI microarray profiles to increase sample size.

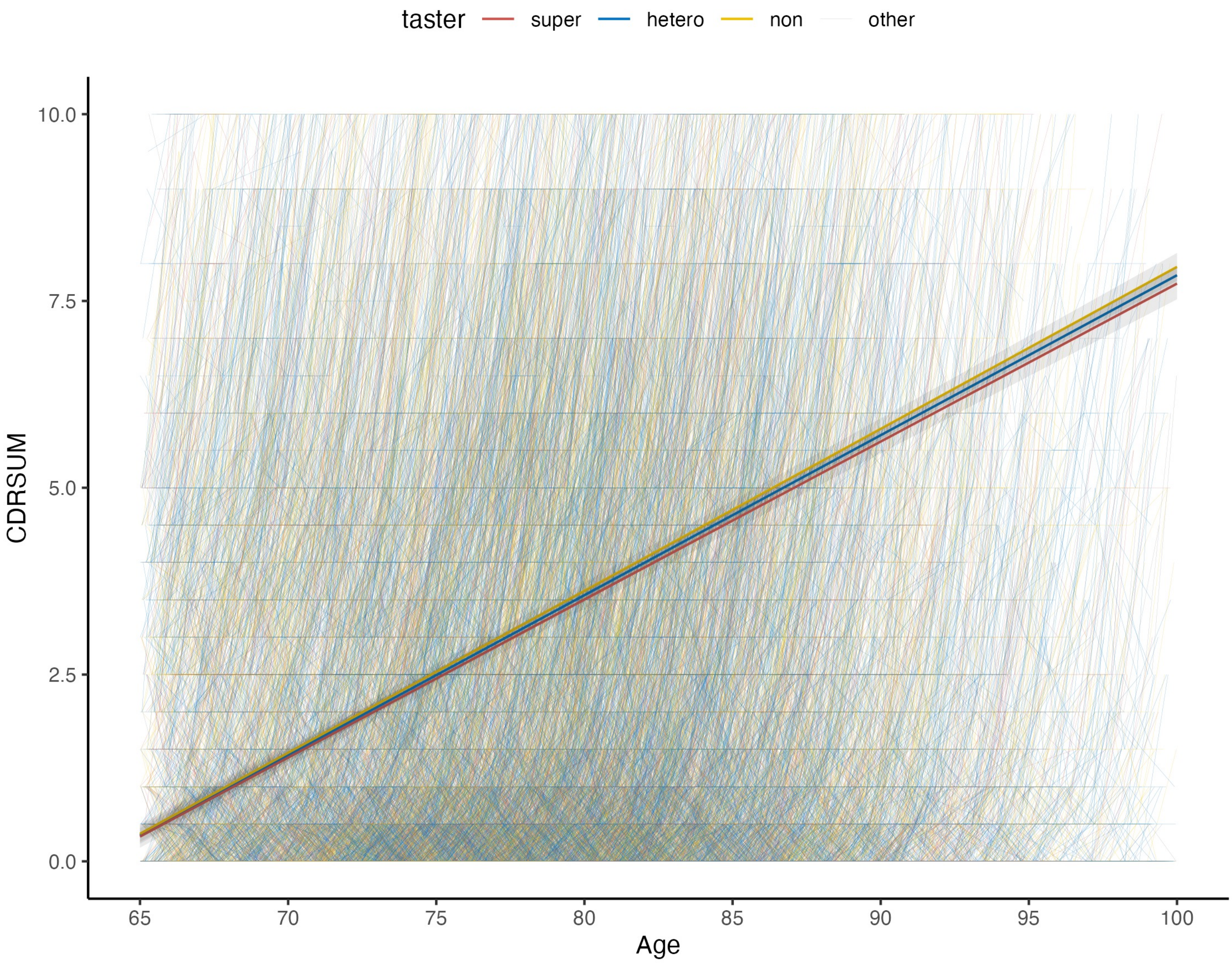

P value	SEX	EDUC	APOE4	age	g1	g2	g3	age:g1	age:g2	age:g3
CDRSUM	<2.2E-16	<2.2E-16	<2.2E-16	2.57E-12	1.14E-08	2.66E-06	1.36E-03	1.82E-09	4.90E-07	8.18E-04
CDRGLOB	<2.2E-16	<2.2E-16	<2.2E-16	4.56E-11	4.34E-08	1.93E-05	5.88E-04	1.16E-08	5.50E-06	4.46E-04
MEMORY	<2.2E-16	<2.2E-16	<2.2E-16	1.36E-11	7.14E-07	5.29E-06	1.76E-02	2.39E-07	1.77E-06	1.27E-02
ORIENT	<2.2E-16	<2.2E-16	<2.2E-16	1.37E-10	1.92E-06	6.24E-05	6.78E-03	5.40E-07	1.71E-05	4.85E-03
JUDGMENT	<2.2E-16	<2.2E-16	<2.2E-16	2.09E-11	3.49E-05	1.07E-03	2.92E-02	1.63E-05	5.44E-04	2.33E-02
HOMEHOBB	<2.2E-16	<2.2E-16	<2.2E-16	1.54E-11	1.61E-06	4.61E-04	1.95E-03	3.87E-07	1.37E-04	1.18E-03
COMMUN	<2.2E-16	<2.2E-16	<2.2E-16	2.63E-11	3.58E-07	3.13E-04	6.12E-04	7.61E-08	8.29E-05	3.67E-04
PERSCARE	<2.2E-16	<2.2E-16	<2.2E-16	3.76E-09	8.26E-04	1.28E-02	2.11E-02	2.94E-04	5.76E-03	1.44E-02
**Effects**	**SEX (F)**	**EDUC**	**APOE4**	**age**	**g1**	**g2**	**g3**	**age:g1**	**age:g2**	**age:g3**
CDRSUM	-0.74370	-0.17820	1.89300	0.12500	-5.06300	-3.32800	1.89100	0.06850	0.04588	-0.02530
CDRGLOB	-0.13750	-0.02997	0.31060	0.02044	-0.84240	-0.52510	0.35210	0.01130	0.00720	-0.00461
MEMORY	-0.14500	-0.03191	0.37440	0.02087	-0.75910	-0.55700	0.24210	0.01017	0.00753	-0.00325
ORIENT	-0.10070	-0.03087	0.34960	0.02058	-0.75640	-0.50810	0.28650	0.01025	0.00704	-0.00382
JUDGMENT	-0.14450	-0.03273	0.31510	0.02186	-0.66960	-0.42270	0.23490	0.00897	0.00576	-0.00313
HOMEHOBB	-0.13380	-0.03301	0.32380	0.02347	-0.82730	-0.48250	0.35580	0.01127	0.00678	-0.00478
COMMUN	-0.12420	-0.03089	0.30280	0.02138	-0.80960	-0.45800	0.36290	0.01100	0.00645	-0.00484
PERSCARE	-0.09569	-0.02067	0.21220	0.02046	-0.57280	-0.34050	0.26340	0.00802	0.00489	-0.00360

Supplementary Figure 2: Longitudinal changes in CDR sum of boxes, with fitted lines stratified by different taster groups in NACC. P values and effect sizes for all the models are reported at the bottom.

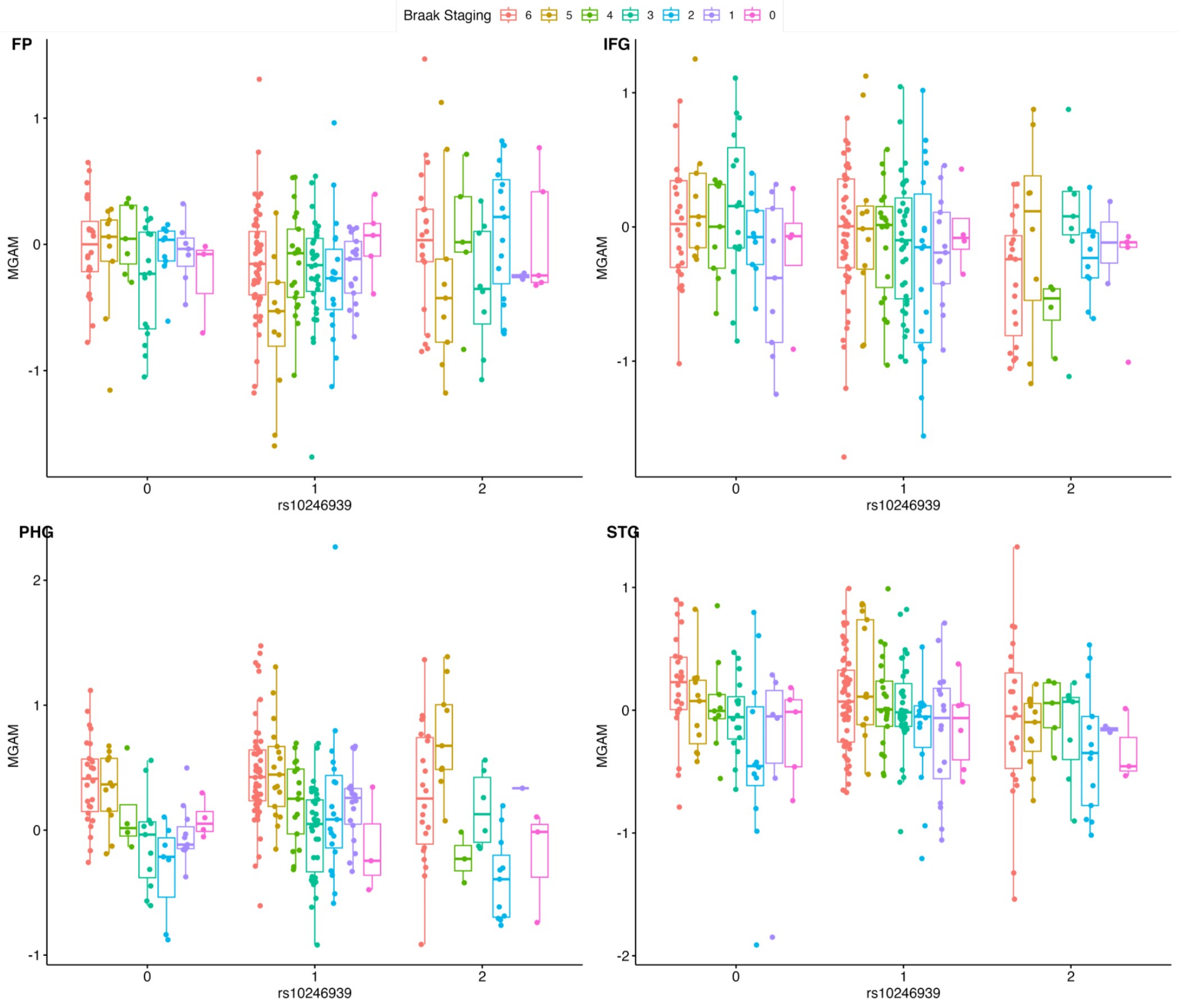

Supplementary Figure 3: MGAM expression split by rs10246939 (g1) alternative allele count and Braak staging from MSBB cohort for four different brain regions.

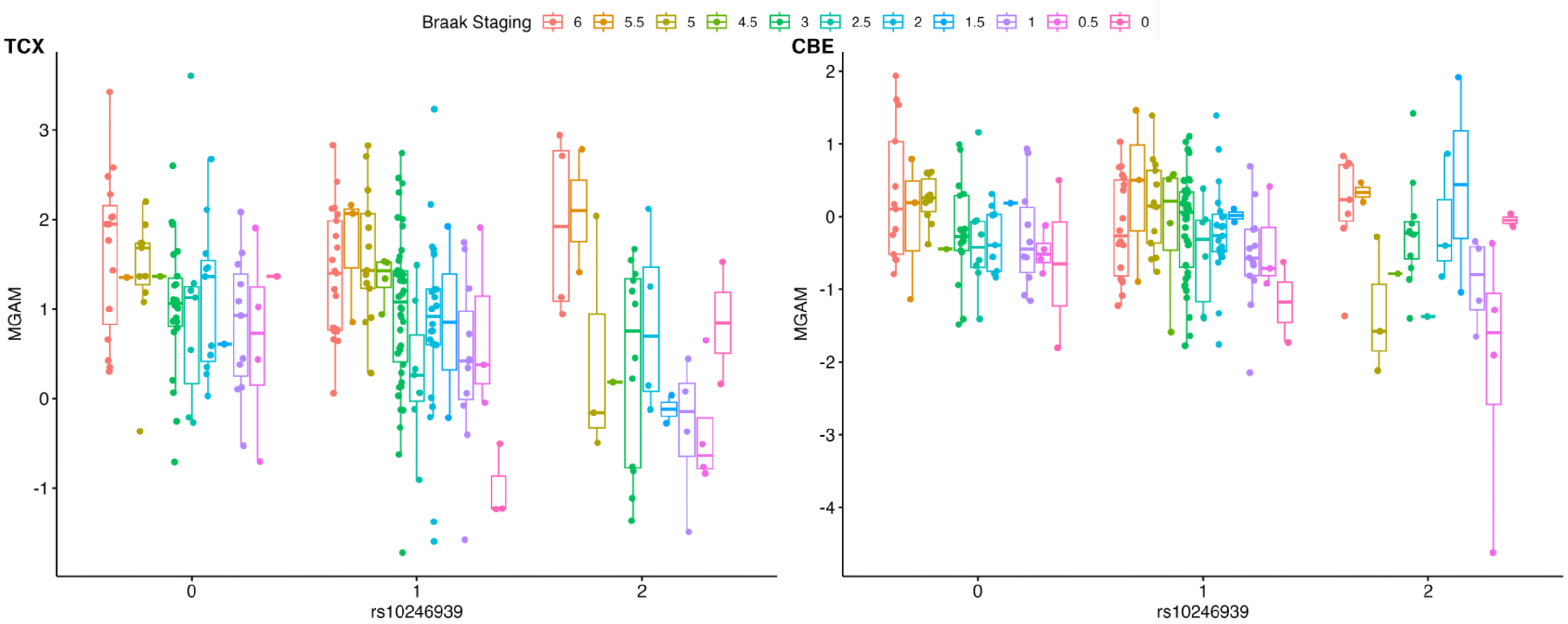

Supplementary Figure 4: MGAM expression split by rs10246939 (g1) allele count and Braak staging from MAYO cohort for two different brain regions.

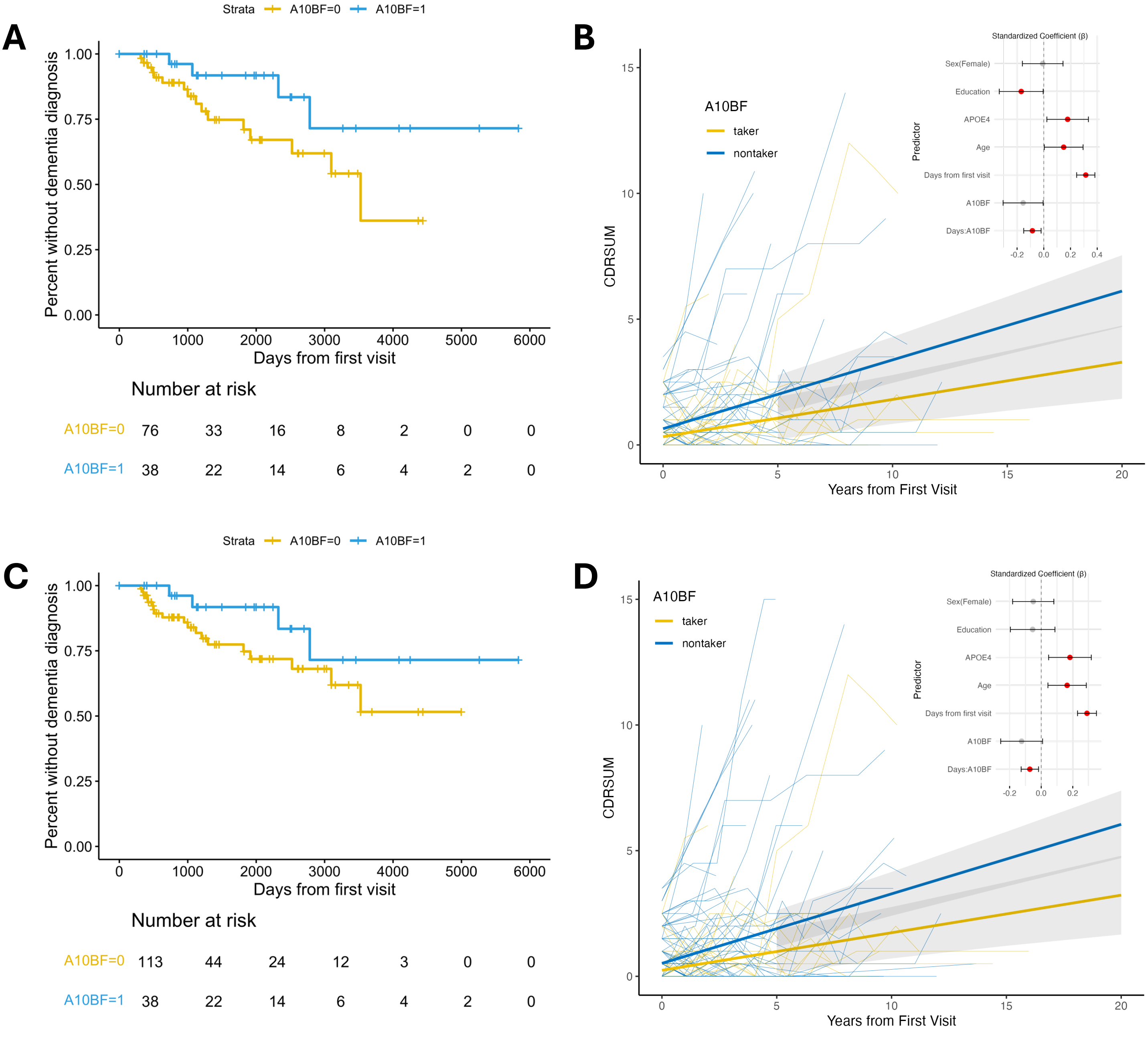

Supplementary Figure 5: MGAM inhibitors slow down cognitive decline in T2D patients in PSM groups. A) Kaplan-Meier curve comparing dementia-free probability between ever users and never users of MGAM inhibitors in PSM groups at ratio=2. N = 38 x 3, P = 0.062. B) Longitudinal changes in clinical assessment (CDRSUM) with fitted lines stratified by drug user groups in A). Group difference P = 0.010. Inset is standardized coefficients for the LME model. Significant terms are colored in red. C) same as A) with ratio = 3, N = 38 x 4 – 1 due to exact match, P = 0.14. D) same as B with ratio = 3, P = 0.010.
